# Anterior ischemic optic neuropathy precipitated by acute primary angle closure

**DOI:** 10.4103/0301-4738.67070

**Published:** 2010

**Authors:** Nikhil S Choudhari, Ronnie George, Vardhman Kankaria, G T Sunil

**Affiliations:** Department of Glaucoma, Jadhavbhai Nathamal Singhvi, Medical Research Foundation, Sankara Nethralaya, 18, College Road, Chennai-600 006, India

**Keywords:** Angle closure, glaucoma, optic neuropathy

## Abstract

A 59-year-old man with a history of longstanding systemic hypotension developed asymmetric non-arteritic anterior ischemic optic neuropathy (NAION) apparently precipitated by bilateral sequential acute primary angle closure. NAION is very rarely reported in association with raised intraocular pressure. In contrast to optical coherence tomography, the failure of scanning laser polarimetry to detect axonal swelling was another interesting finding. Possible reasoning for these observations is discussed.

Nonarteritic Anterior Ischemic Optic Neuropathy (NAION) is a result of circulatory insufficiency within the Optic Nerve Head (ONH). An anatomically small optic disc with a correspondingly smaller optic cup resulting in ’crowding’ of nerve fibers at the ONH is believed to play a causative role.[1] Arteriosclerosis, vasospasm or systemic hypotension leading to impairment in the normal autoregulatory mechanisms of the ONH is another postulated mechanism to explain NAION.[2] Raised intraocular pressure (IOP) can also decrease perfusion pressure at the ONH. But NAION in the setting of acute primary angle closure (APAC) is rarely reported in the literature.[3–6] The purpose of reporting this case is to demonstrate evolution of NAION following APAC.

## Case Report

A 59-year-old gentleman was seen with a history of sudden onset pain, redness and diminution of vision of three weeks duration in the right eye (RE) and a week’s duration in the left eye (LE). He had an associated history of abdominal pain, nausea and vomiting. He also gave a history of longstanding systemic hypotension.

At presentation, he had visual acuities of 20/400 (RE), and 20/120 (LE). He had corneal edema and a shallow peripheral anterior chamber in both eyes with mid-dilated sluggish pupils. There were glaucomflecken in LE. The applanation pressures measured 44 mmHg (RE) and 46 mmHg (LE). He was treated with intravenous Mannitol 250 mL, Tablet Acetazolamide (250 mg) four times a day, and topical Prednisolone Acetate one-hourly, Timolol Maleate (0.5%) twice a day, Pilocarpine (2%) four times a day in both eyes (BE) after which corneal edema subsided. Gonioscopy confirmed bilateral angle closure. He underwent peripheral YAG laser (VISULAS YAG II, Carl Zeiss Meditec, Germany)-assisted iridotomy in both eyes (BE). Topical aqueous suppressants were continued.

On the subsequent day, applanation pressures were 10 (RE) and 7 mmHg (LE). A relative afferent pupillary defect (RAPD) was evident in LE. Post-iridotomy gonioscopy revealed 360-degree peripheral anterior synechiae in BE. The right optic disc was moderate in size (vertical disc diameter 1.8 mm) with a pink neuroretinal rim and had a vertical cup-disc ratio of 0.4:1 while the left optic disc (approximate vertical disc diameter 1.8 mm) showed pallid edema [Fig. 1A,B]. Color vision recording with Ishihara plates were 14/14 in both eyes. He was found to have visual field constriction in RE and a tubular field in LE (SITA Standard 30-2 program, Humphrey perimeter, Carl Zeiss Meditec, Dublin, CA, Fig. 2). Fundus fluorescein angiography (FFA) showed choroidal defects and hypo-perfusion of the nasal half of the left optic disc [Fig. 3]. There was significantly increased retinal nerve fiber layer (RNFL) thickness in LE on Optical Coherence Tomography (Stratus OCT, Carl Zeiss Meditec, Germany). However, RNFL thickness with Scanning Laser Polarimetry (SLP [GDx VCC, Carl Zeiss Meditec, Germany]) was almost within the normal range in BE. His physical evaluation was unremarkable with a pulse rate of 60/min and blood pressure of 110/70 mmHg. His complete hemogram including erythrocyte sedimentation rate was normal.

**Figure 1A:**
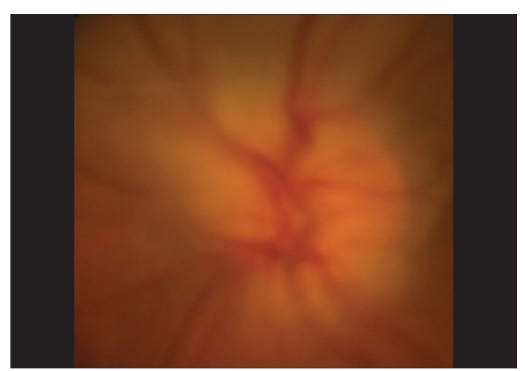
Color photograph of the left optic disc following resolution of acute angle closure

**Figure 1B:**
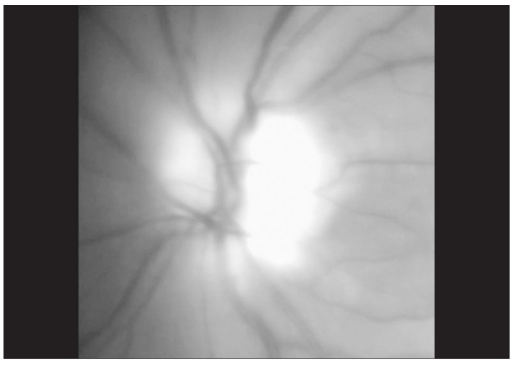
Red-free photograph of the left optic disc following resolution of acute angle closure

**Figure 2:**
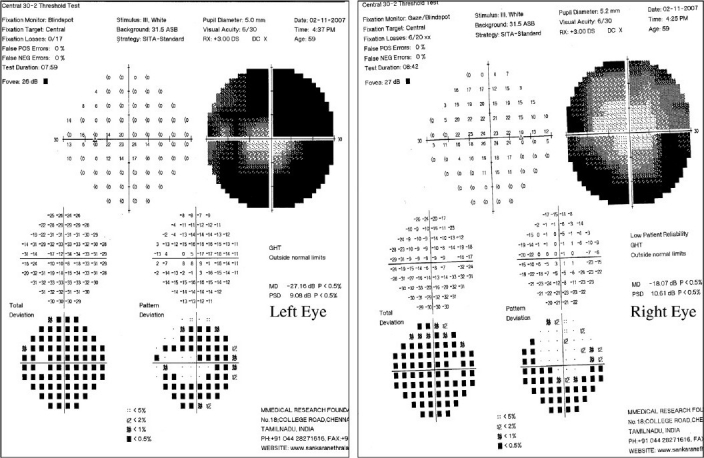
Humphrey visual field, Program 30-2 printouts of left and right eye

**Figure 3:**
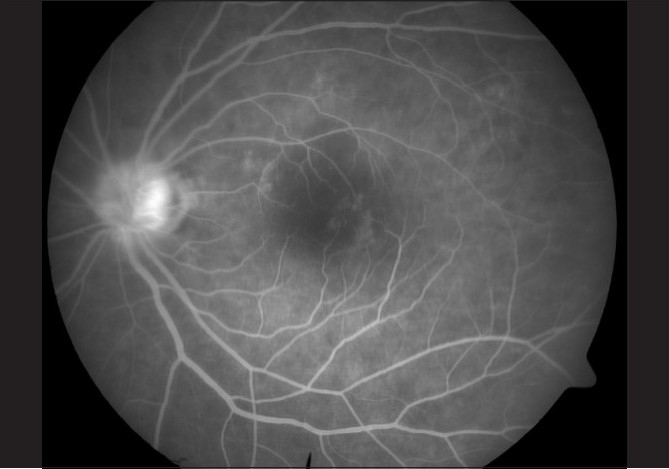
Late arterio-venous phase on fundus fluorescein angiography of the left eye

**Figure 4:**
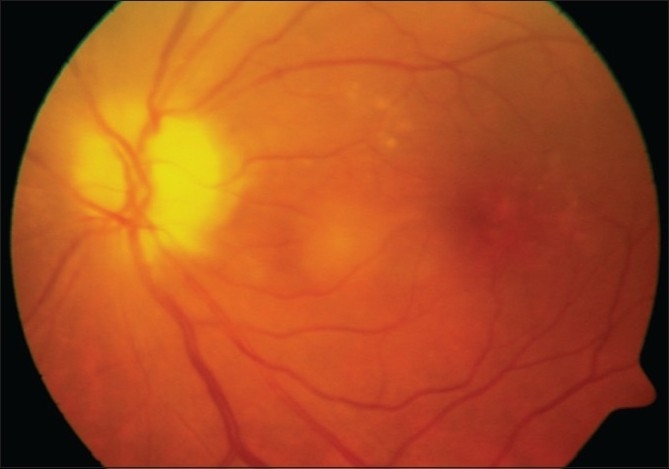
One month follow-up fundus photograph of the left eye

On subsequent follow-up a month later, the patient had no new symptoms. His visual acuities were 20/30 in RE and 20/40 in LE. The applanation pressures measured 13 (RE) and 12 (LE) mmHg. The right optic disc showed mild diffuse pallor and the left optic disc showed gross pallor with complete resolution of edema [Fig. 4]. Three months later, his visual acuities were 20/20 both eyes, IOPs were in low teens on topical anti-glaucoma medications, both optic discs had pallor (left > right) and there was no significant change in his visual fields.

## Discussion

NAION is presumed to be a result of hypo-perfusion within the ONH.[2] A significant rise in IOP can cause reduction in the perfusion pressure of the ONH from compression of vessels in the prelaminar region and can lead to NAION. The choroidal contribution to the blood supply of the optic disc and the peripapillary choroid have been shown to be the most susceptible to obliteration by the elevated IOP.[7] The appearance of clinical ONH ischemia is related to the maximum IOP recorded and the time required to reach the peak.[8] In our case, even though the duration of symptoms was shorter in LE and the IOPs at presentation were similar in BE, the sequels of APAC were more severe in LE. This may mean that LE had higher initial IOP or had taken a shorter duration than RE to reach the peak level of IOP. NAION in LE was indicated by a significant pallor of the disc without loss of neuroretinal rim, constriction of visual field as opposed to (bi) arcuate pattern (nerve fiber bundle type) of visual field loss, and choroidal perfusion defects and hypo-perfusion of the nasal half of the optic disc on FFA. A low sensitivity of Ishihara chart in detecting red green color defects is known.[9] An alternate cause for the normal Ishihara recording could be preservation of color vision in the relatively normal central field in LE. The reason for the constriction of visual field in RE is unclear. We postulate the occurrence of subclinical optic nerve ischemia indicated by the subsequent appearance of mild, diffuse pallor of the right optic disc.

Optic disc edema and hemorrhages have been reported following acute glaucoma.[8] However, considering the incidence of APAC, the reports where ischemic optic neuropathy appears to be precipitated by APAC are scarce.[3–6] The principle of increased IOP in reducing optic nerve perfusion simply does not explain why NAION is so rarely associated with increased IOP even if media opacity during acutely increased IOP is considered. Other factors that compromise ONH circulation might have to be operational for the NAION to manifest in the setting of acute angle closure. In this regard, we give importance to the history of a longstanding systemic hypotension in our patient, even though we could not confirm it.

SLP measures form birefringence properties to estimate RNFL thickness.[10] In contrast, OCT is comparatively a more direct measure of RNFL thickness.[10] Obstruction of axonal transport in the region of the lamina cribrosa is associated with axonal swelling and an accumulation of mitochondrial aggregates but not necessarily microtubules, which are the dominant source of RNFL birefringence.[10] Our findings reemphasize the fact that OCT, rather than SLP should be considered to monitor optic disc edema over time. To conclude, ischemic optic neuropathy can be precipitated by APAC, and other factors that compromise ONH circulation might be operational in this setting.
